# A peptidoglycan hydrolase motif within the mycobacteriophage TM4 tape measure protein promotes efficient infection of stationary phase cells

**DOI:** 10.1111/j.1365-2958.2006.05473.x

**Published:** 2006-12-02

**Authors:** Mariana Piuri, Graham F Hatfull

**Affiliations:** Pittsburgh Bacteriophage Institute and Department of Biological Sciences, University of Pittsburgh Pittsburgh, PA 15260, USA

## Abstract

The predominant morphotype of mycobacteriophage virions has a DNA-containing capsid attached to a long flexible non-contractile tail, features characteristic of the Siphoviridae. Within these phage genomes the tape measure protein (*tmp*) gene can be readily identified due to the well-established relationship between the length of the gene and the length of the phage tail – because these phages typically have long tails, the *tmp* gene is usually the largest gene in the genome. Many of these mycobacteriophage Tmp's contain small motifs with sequence similarity to host proteins. One of these motifs (motif 1) corresponds to the Rpf proteins that have lysozyme activity and function to stimulate growth of dormant bacteria, while the others (motifs 2 and 3) are related to proteins of unknown function, although some of the related proteins of the host are predicted to be involved in cell wall catabolism. We show here that motif 3-containing proteins have peptidoglycan-hydrolysing activity and that while this activity is not required for phage viability, it facilitates efficient infection and DNA injection into stationary phase cells. Tmp's of mycobacteriophages may thus have acquired these motifs in order to avoid a selective disadvantage that results from changes in peptidoglycan in non-growing cells.

## Introduction

All bacteriophages are posed with the common problem of how to accomplish injection of their genomes across the walls and membranes of their host bacterial cells (Molineux, 2001; 2006). Phages that infect Gram-negative bacteria are faced with two membrane layers, but with only a relatively thin peptidoglycan layer within the periplasmic space (Vollmer and Holtje, 2001). Nevertheless, the virion particles of several phages of Gram-negative hosts have been shown to contain hydrolytic activities targeted against peptidoglycan; for example, virions of T4, T7, ϕ6 and PRD1 contain either lysozyme, transglycosylase or endopeptidase activities (Nakagawa *et al*., 1985; Caldentey and Bamford, 1992; Moak and Molineux, 2000; Rydman and Bamford, 2000; Kanamaru *et al*., 2002). The location of these functions within the virions is varied. For example, in phage T4 the lysozyme (gp5) is a component of the baseplate's central hub (Mosig *et al*., 1989), whereas the transglycosylase of T7 (gp16) is one of three proteins present within the capsid (Struthers-Schlinke *et al*., 2000). In ϕ6 the endopeptidase (P5) is located between the nucleocapsid surface and the viral membrane (Caldentey and Bamford, 1992), and in PRD1 both a transglycosylase (P7) and a 1,4 beta-*N*-acetylmuramidase activities (P15) are located within the viral membrane (Rydman and Bamford, 2002). While the specific roles of these peptidoglycan-degrading activities may differ somewhat, it is likely that they share a common function in facilitating efficient infection of host cells, either by enabling access to the cytoplasmic membrane or mediating transfer of virion DNA from the capsid to the cellular cytoplasm.

Phages that infect Gram-positive hosts have only a single cytoplasmic membrane to contend with, but the peptidoglycan layer is usually much thicker than in Gram-negative bacteria. Not surprisingly, the presence of muralytic activities in the virions of such phages (including those that infect *Lactococcus lactis*, *Staphylococcus aureus* and *Bacillus subtilis*) is not uncommon (Moak and Molineux, 2004), and in the *L. lactis* phage Tuc2009 there is a tail-associated lysin involved in localized cell wall degradation (Kenny *et al*., 2004). Phages that infect mycobacterial hosts must not only overcome the membrane and peptidoglycan barriers but must also contend with thick complex cell walls that contain long carbon chain mycolic acids (Brennan, 2003). However, virion-associated enzymatic activities that degrade peptidoglycan, mycolic acids or other cell wall components have yet to be identified. Interestingly, while the virion morphologies of many mycobacteriophages have been examined, all contain either a contractile tail (Myoviridae) or a long flexible non-contractile tail (Siphoviridae), and to our knowledge no short-tailed phages (e.g. Podoviridae) have been isolated. As short-tailed phages are prevalent among phages that infect a variety of other bacterial hosts, the question arises as to whether longer phage tails are needed to traverse complex mycobacterial cell walls.

A comparative genomic analysis of 14 mycobacteriophages reveals that several contain unusual sequence motifs embedded within their tape measure proteins (Tmp's) (Pedulla *et al*., 2003). Three motifs were identified (motifs 1, 2 and 3) all of which have sequence similarity to previously identified host proteins that are not associated with phage or prophage genomes. Motif 1 (present in mycobacteriophage Barnyard) is related to a family of proteins typified by the Resuscitation Promoting Factor (Rpf) of *Micrococcus luteus* but found in many other bacteria (Mukamolova *et al*., 2002), motif 2 (present in mycobacteriophages Che9c, Cjw1, Barnyard and Rosebush) is related to a small *Mycobacterium tuberculosis* protein of unknown function (Rv1115), and motif 3 (mt3, present in TM4, Che8 and Omega) is related to two small *M. tuberculosis* proteins Rv0320 and Rv1728c (Pedulla *et al*., 2003). The role of these motifs has remained unclear, although their presence within the Tmp's of these phages is somewhat surprising because these proteins have been previously shown to play an important role in phage tail assembly, such that there is a direct correlation between the length of the *tmp* gene and the length of the phage tail (Katsura and Hendrix, 1984). Indeed, small deletions and duplications within the lambda *tmp* gene (*H*) yields phage particles with correspondingly shorter or longer tails (Katsura and Hendrix, 1984; Katsura, 1987); a similar mutational analysis has been performed with lactococcus phage TP901-1 with similar conclusions (Pedersen *et al*., 2000). The presence of the small sequence motifs suggests that Tmp's may play important roles other than in tail assembly.

Recently, it has been demonstrated that the Rpf-related proteins have structural similarity to lysozymes and soluble transglycosylases, and can hydrolyse peptidoglycan *in vitro* (Cohen-Gonsaud *et al*., 2005; Keep *et al*., 2006; Telkov *et al*., 2006). However, they were initially identified through the ability of the Rpf protein to resuscitate the growth of dormant *Micrococcus luteus* cells (Mukamolova *et al*., 1998). The peptidoglycan-degrading activity appears to be required for resuscitation (Mukamolova *et al*., 2006) suggesting that this either leads to activation of a cell cycle step, or possibly releases other growth promoting signals from lysed cells (Keep *et al*., 2006). While the role of motif 1 in Barnyard remains unclear, these observations raise the question as to whether motifs 2 and 3 are also involved in the degradation of peptidoglycan or other cell wall components.

In this paper we explore the activity of mt3 present within the tape measure protein of mycobacteriophage TM4 and its homologous proteins encoded by the *Mycobacterium smegmatis* host. We show that the host proteins MSMEG0642, MSMEG3721 and TM4 Tmp mt3 contain peptidoglycan-degrading activity. Removal of mt3 from the TM4 Tmp generates virions with predictably shortened tails but which are still viable. While the parental phage has a similar efficiency of plating on stationary phase cells relative to exponentially growing cells, the efficiency of plating of the mt3 deletion mutant on stationary phase cells is reduced approximately 50% relative to exponentially growing cells; mutant phage with a single amino acid substitution of a well-conserved tryptophan residue in the motif has a similar phenotype. This reduction in plating efficiency is not due to a change in adsorption but as a result of a defect in DNA injection as indicated by the behaviour of luciferase reporter phage derivatives. We conclude that Tmp mt3 confers the ability of TM4 to efficiently infect stationary phase cells that probably have thicker or more highly cross-linked peptidoglycan in their cell walls, and confers a selective advantage over those phages that do not possess this capability.

## Results

### Relationship of TM4 Tmp mt3 to a family of host proteins

**Fig. 1:**
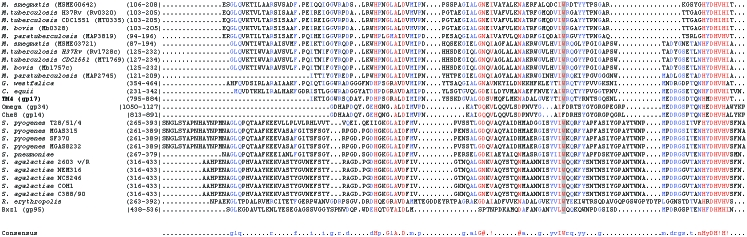
Amino acid sequence alignment of proteins related to Tmp mt3. Amino acid sequence alignment of proteins containing the ProDom domain that corresponds to mt3 using MultAlign. Omega gp34 and Che8 gp14 were included in the alignment using clustalx. Red letters, high consensus (90%); blue letters, low consensus (50%); ! is anyone of IV; # is anyone of NDQE. For Mycobacterial and Mycobacteriophages proteins the correspondent gene name is also shown. The conserved W residue is highlighted.

We showed previously that the conserved sequence mt3 present in the tape measure proteins of mycobacteriophages TM4, Omega, and Che8 is related to *M. tuberculosis* Rv1728c and Rv0320, two putative *M. tuberculosis* hypothetical conserved proteins of unknown function, as well as to a putative peptidase of *Rhodococcus equi* (Pedulla *et al*., 2003*)*. Rv1728c and Rv0320 are highly conserved among sequenced mycobacterial genomes including *M. tuberculosis* CDC 1551, *Mycobacterium bovis* AF2122/97, *Mycobacterium avium* ssp. *paratuberculosis* str K10 and *M. smegmatis* mc^2^155; the *M. smegmatis* homologues, MSMEG3721 and MSMEG0642, share 70% and 58% amino acid sequence identity with *M. tuberculosis* Rv1728c and Rv0320 respectively (Fig. 1). The conserved motif is located at positions 87–194 and 106–208 in MSMEG3721 and MSMEG0642 respectively (Fig. 1).

A protein domain search using ProDom (Servant *et al*., 2002; Bru *et al*., 2005) revealed that TM4 Tmp mt3 is present as a domain within at least two dozen bacterial proteins (Fig. 1). The functions of these have not been experimentally demonstrated, although several have been annotated as having either a role in cell wall catabolism or functioning as metalloendopeptidases, mainly because of the presence of other sequence motifs (e.g. LysM, VanY, Transglycosylas) found in cell wall enzymes. A role for the members of this family in cell wall catabolism is supported by the bioinformatic prediction that all of the bacterial proteins including MSMEG3721, MSMEG0642, Rv1728c and Rv0320 contain putative signal sequences as well as putative processing sites (at positions 25/66 in MSMEG3721/Rv1728c and 40/38 in MSMEG0642/Rv0320 respectively). Members of the family also include zinc metalloendopeptidases, which can act lytically against bacterial cells (Li *et al*., 1998; Nilsen *et al*., 2003).

The output from ProDom includes the Tmp of phage TM4, as well as a protein (gp95) encoded by mycobacteriophage Bxz1 whose virions possess a contractile tail (Pedulla *et al*., 2003). However, it does not includes the Tmp's of mycobacteriophages Omega and Che8 that were previously described as including mt3 (Pedulla *et al*., 2003), even though these can be readily included in the alignment using clustalx. A plausible explanation is that Omega and Che8 Tmp's do not possess a well-conserved isoleucine-tryptophan sequence at the centre of the motif, where the tryptophan is substituted with phenylalanine (Fig. 1).

### MSMEG3721 and MSMEG0642 are peptidoglycan-degrading enzymes

To address whether this family of proteins is active in cell wall catabolism we have taken three approaches to characterize the host proteins MSMEG3721 and MSMEG0642. First, we examined the cellular localization of MSMEG3721 in *M. smegmatis*, because cell wall enzymes are likely to be located on the exterior of the cell. To monitor the expression and localization of the protein we overexpressed and purified it from *Escherichia coli* cells and raised polyclonal antibodies. Surprisingly, although these antibodies are of respectable titer and readily recognize both purified protein and MSMEG3721 expressed in *E. coli* (data not shown), we were unable to detect expression of the endogenous protein in either exponentially growing or stationary phase cells of *M. smegmatis* (data not shown).

**Fig. 2:**
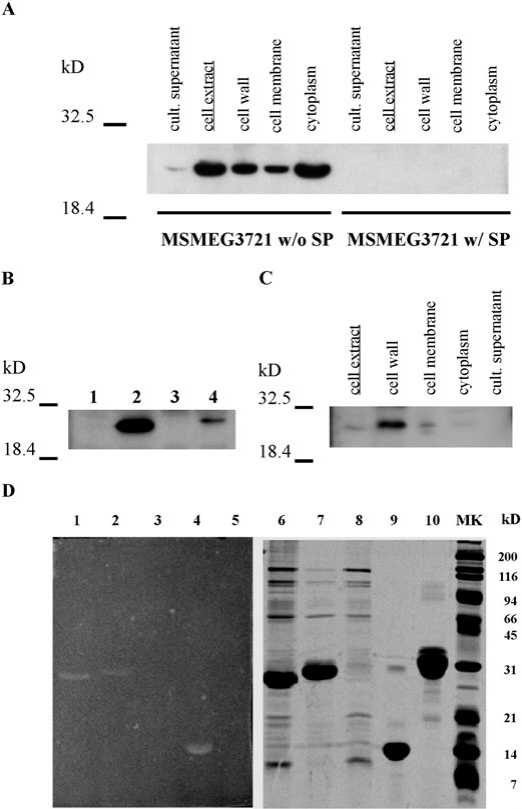
Localization and biochemical activities of MSMEG3721/MSMEG0642. A. Western blot analysis of MSMEG3721 with or without the putative SP overexpressed in *M. smegmatis* using an extrachromosomal plasmid (pMP2 and pMP1 respectively). Samples of cell fractions are indicated. B. MSMEG3721 gene containing the putative SP was cloned under control of an acetamidase promoter in an integrative vector (pMP4). After integration in *M. smegmatis*, cells were grown in TH9 containing succinate with and without the acetamide inducer. Proteins were extracted from *M. smegmatis* cells transformed with pJL37 (empty vector control; lane 1), pMP2 (control of expression w/out SP; lane 2), pMP4 (grown in the absence of acetamide; lane 3), pMP4 (grown in the presence of acetamide; lane 4). C. Cell localization of MSMEG3721 w/SP overexpressed from pMP4 in the presence of acetamide. In all cases proteins were detected in the indicated cell fractions using an α-MSMEG3721 polyclonal antiserum. D. Partially purified protein preparations were separated by SDS-PAGE (right panel) or separated and analysed in a zymogram (left panel) for peptidoglycan-hydrolysing activity. Lanes 1 and 6, MSMEG3721 recombinant protein (10 μg); 2 and 7 MSMEG0642 recombinant protein (10 μg); lanes 3 and 8, mock purification using *E. coli* cells transformed with pET-28a (empty vector); lanes 4 and 9 lysozyme (5 μg) used as positive control; lanes 5 and 10, β-casein (10 μg) used as a negative control.

If MSMEG3721 is involved in cell wall catabolism it is perhaps not surprising that the protein is expressed at only very low levels. In order to circumvent this detection problem we attempted to elevate the expression by cloning the gene into extrachromosomal plasmid vectors containing the strong *M. bovis* BCG *hsp60* promoter (Stover *et al*., 1991), and constructed plasmid derivatives containing the gene both with and without its putative signal peptide (SP). Interestingly, while the protein lacking the SP was readily detected and is primarily located within the cytoplasm, we were not able to detect any protein expression from the full-length gene (Fig. 2A). Presumably, the protein is either not expressed or is processed and rapidly degraded; it is unlikely that it is secreted because we do not detect protein in the culture supernatant (Fig. 2A). However, when expression of the protein was induced from the regulatable acetamidase promoter (Parish *et al*., 1997), we could detect small amounts of the protein expressed with SP (Fig. 2B). Fractionation of the induced cells shows that the protein is primarily located within the cell wall fraction (Fig. 2C); we could not detect any of this protein in spent growth medium (Fig. 2C).

To further elucidate the possible functions of MSMEG0642 and MSMEG3721 we constructed knockout mutants for each gene individually and with both together. None of the three mutants showed any obvious growth defect compared with the parent strain (data not shown). Presumably, either they do not provide functions essential for growth or there is an additional gene that is functionally redundant to these. We note that in *E. coli* a mutant lacking three known endopeptidases (PBPs 4 and 7, and MepA) is viable, presumably for similar reasons (Young, 2001). However, as MSMEG0642 and MSMEG3721 could also be candidates for peptidoglycan hydrolases, we decided to test whether the mutants exhibited any changes to antibiotics that are known to act on peptidoglycan biosynthesis. Interestingly, we found that all three of the mutants show a modest but significant increase in resistance to vancomycin (Table 1 and Fig. S1), an antibiotic that binds to the d-alanyl-d-alanine peptide terminus of the nascent peptidoglycan-lipid carrier, is incorporated into nascent peptidoglycan polymer, and prevents full cross-linking by the transpeptidases. This increase in resistance suggests that the peptidoglycan is altered in these mutant strains, consistent with the hypothesis that MSMEG0642 and MSMEG3721 are involved in peptidoglycan synthesis or remodelling. Complementation studies are consistent with the interpretation that the loss of MSMEG0642 and/or MSMEG3721 functions contributes to vancomycin resistance although the data are complex because a restoration of normal drug sensitivities is only observed when the complementing gene is provided on an extrachromosomal plasmid (Table 1), and not when on an integration-proficient plasmid (data not shown). Finally, we note that a mutant of a known *M. smegmatis* peptidoglycan endopeptidase (PBP4, MSMEG6076, a putative d-alanyl-d-alanine carboxypeptidase/d-alanyl-d alanine-endopeptidase) is also resistant to vancomycin and could thus be functionally redundant to MSMEG0642 and MSMEG3721 (Table 1).

**Table 1:** Minimal inhibitory concentration (MIC) for vancomycin of wt and mutant strains.

Strain	Vancomycin MIC (μg ml^−1^) ± SD	R factor*
Wild type	2.43 ± 0.04	1
MSMEG3721:: *res-hyg-res*	5.26 ± 0.20	2.2
MSMEG0642:: *res-hyg-res*	4.83 ± 0.09	2.0
MSMEG0642Δ/3721:: *res-hyg-res*	4.99 ± 0.18	2.1
MSMEG6076:: res-hyg-res	12.41 ± 0.70	5.1
*wt* (pPG1)	4.64 ± 0.04	1
MSMEG0642Δ/3721:: *res-hyg-res* (pPG1)	8.75 ± 0.03	1.9
MSMEG0642Δ/3721:: *res-hyg-res* (pMP8)	4.51 ± 0.09	1
MSMEG0642Δ/3721:: *res-hyg-res* (pMP9)	4.72 ± 0.16	1

Experiments were done by triplicate and the mean for the MIC and the SD are shown.

* R factor represents the resistance factor of each strain compared with *M. smegmatis* mc^2^155 (wild type).

To more directly assess the enzymatic activity of MSMEG0642 and MSMEG3721 we tested their ability to generate a zone of clearing in a zymogram assay. In this assay proteins are separated by SDS gel electrophoresis through a matrix containing peptidoglycan, renatured, and allowed to degrade the peptidoglycan; the peptidoglycan is then stained with methylene blue (Moak and Molineux, 2004). Proteins capable of degrading peptidoglycan generate a white zone of clearing on a dark background. As shown in Fig. 2D, both MSMEG3721 and MSMEG0642 are positive in this assay. While the specific activity of these proteins appears to be lower than that of a lysozyme control (perhaps four- to fivefold reduced), relative activities must be interpreted cautiously because enzyme renaturation efficiencies can be highly variable (Fig. 2D).

### TM4 gp17 mt3 supports peptidoglycan hydrolytic activity

**Fig. 3:**
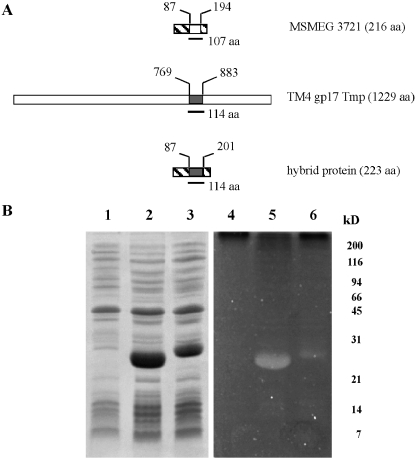
Construction of a hybrid protein carrying TM4 Tmp mt3. A. Schematic representation of TM4 gp17 Tmp and MSMEG3721 showing the position of mt3. A hybrid protein was created by replacing mt3 in MSMEG3721 with the motif present in the Tmp of TM4. B. *E. coli* cell extracts expressing the indicated proteins were separated by SDS-PAGE (left panel) or separated and analysed in a zymogram for peptidoglycan-hydrolysing activity (right panel). Lanes 1 and 4, extract of *E. coli* cells transformed with pET-28a (empty vector); lanes 2 and 5, *E. coli* cell extracts overexpressing MSMEG3721; lanes 3 and 6, *E. coli* cell extracts overexpressing the hybrid protein.

The analysis described above presents a strong case that MSMEG3721 and MSMEG0642 are involved in cell wall catabolism. As mycobacteriophage TM4 is clearly related to these proteins through the presence of mt3 in the Tmp (Fig. 1), we tested whether TM4 gp17 is active in a zymogram assay. While we were unable to detect any activity from whole phage particles (data not shown) it is plausible that the relatively low abundance and large size of the protein (1229 amino acids) interferes with efficient refolding of the protein within the gel. As attempts to express either the complete TM4 gp17 protein or parts of it in *E. coli* failed, we constructed a hybrid protein where the resident motif (107 residues of the 216 total amino acids) in MSMEG3721 was replaced by mt3 of TM4 gp17 (114 residues) (Fig. 3A); this hybrid protein supports peptidoglycan hydrolysis in a zymogram assay (Fig. 3B). As we have not been able to successfully overexpress a MSMEG3721 derivative in which the conserved motif is deleted, we cannot eliminate the possibility that the peptidoglycan-hydrolysing activity resides elsewhere in the MSMEG3721-derived portion of the hybrid protein. However, as the longest segment of MSMEG3721-derived sequence is only 62 amino acids (see *Experimental procedures*), and bioinformatic analysis does not reveal any conserved sequence motifs other than that described above, we prefer the parsimonious explanation that the TM4 Tmp mt3 provides the catalytic residues.

### Construction of mutant TM4 phages lacking functional mt3's in their Tmp's

**Fig. 4:**
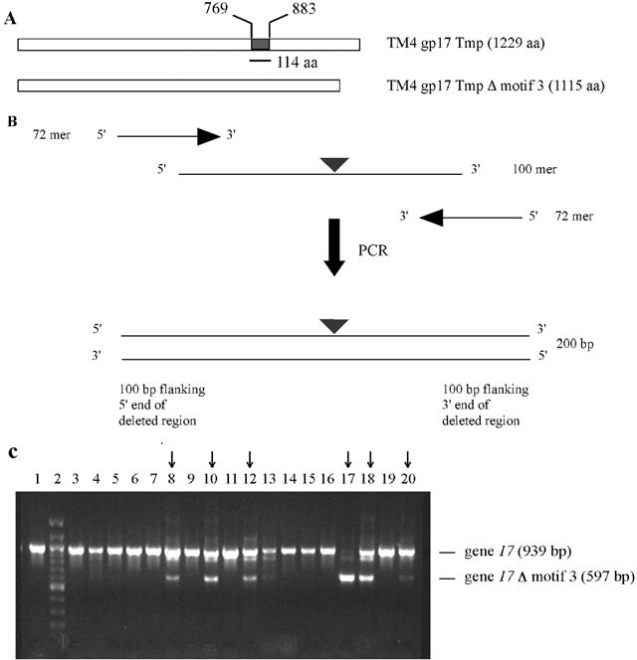
Construction of TM4 deletion mutant lacking Tmp mt3. A. Schematic representation of TM4 gp17 Tmp showing the position of mt3 and the expected product after its deletion. B. Schematic representation of the PCR method used to increase the length of homology in the targeting vector. A 200 bp targeting fragment was obtained from a 100-mer oligonucleotide with 50 bases of homology to each side of the deletion and two 72-mers to generate a 342 bp deletion in TM4 gene *17* (adapted from Swaminathan *et al*., 2001). C. Identification of mutant clones by PCR. Phasmids extracted from pools of cells recovered without selection (approximately three cells/pool) were analysed by PCR using primers that distinguish wild-type and mutant forms of gene *17* and the products analysed by agarose gel electrophoresis; the amplicon obtained from *wt* gene *17* is 939 bp and from gene Δ*17* mt3 is 597 bp. Lanes 3–20 contain 18 representative pools and those containing the phAE87Δ*17*mt3 mutant are marked with an arrow; lane 1 is a parental phAE87 control template and lane 2 is a DNA size marker. Of the total 93 pools tested, 23 contained at least one deletion derivative, and we estimate that the frequency of mutants in the recovered cells is about one recombinant per 12 electroporated cells.

**Fig. 5:**
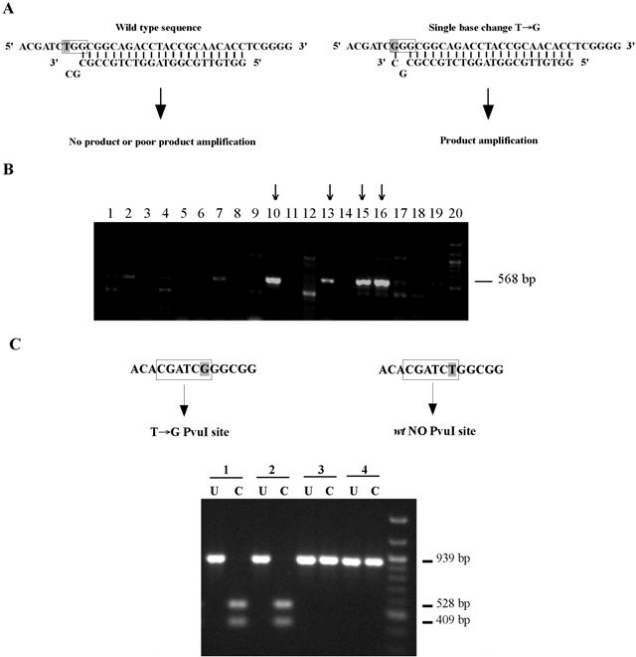
Construction of a point mutant in TM4 Tmp mt3. To construct a point mutation in the shuttle phasmid phAE87, two complementary single-strand oligonucleotides (100-mer) containing a T→G substitution that changes a TGG for a GGG codon (W853G) in gene *17* were electroporated into an *E. coli* recombineering strain and pools of recovered cells screened first by allele-specific PCR amplification (MAMA-PCR) (Cha *et al*., 1992), and then by diagnostic restriction digestion. A. Schematic representation of MAMA-PCR. A detection primer (reverse primer) with a two-base mismatch at the 3′ does not efficiently amplify the wild-type sequence under the PCR conditions used (see *Experimental procedures*), although the same primer anneals with the mutated sequence and effectively amplifies a 568 bp product. B. PCR to identify positive clones after recombineering. Pools of recombineering products (approximately five cells/pool) were analysed by MAMA-PCR and the products analysed by agarose gel electrophoresis. Lanes 2–19 contain representative pools, and those containing phasmids with the desired single base substitution T→G (phAE87*17*W853G) are indicated with an arrow. Lane 1 contains parent control DNA and lane 20 is a DNA marker. Of the 93 pools tested, 25 contained at least one mutant derivative, corresponding to a frequency of approximately one recombinant per 20 electroporated cells. C. Diagnostic restriction analysis of phAE87*17*W853G. Positive recombineering pools were used to transform XL-Blue cells and individual recovered phasmids used as substrates in a second round of MAMA-PCR. Independently recovered clones were tested by amplification of a 939 bp fragment containing the mutant region of TM4 gene *17*, followed by digestion with Pvu I. The T→G substitution generates a new Pvu I site that is absent in the original sequence so that digestion generates 409 bp and 528 bp fragments. Uncut (U) and cut (C) products of two positive (#1 and 2) and two negative (#3 and 4) clones are shown.

In order to determine the role of the TM4 Tmp motif *in vivo* we constructed TM4 derivatives that either lack the Tmp motif (Fig. 4) or carry a non-functional one (Fig. 5). To construct these mutant derivatives of TM4 we adapted the *E. coli* recombineering system that utilizes a highly efficient recombination system and short DNA substrates (Court *et al*., 2002); as TM4 does not replicate in *E. coli* we used a previously constructed shuttle phasmid phAE87 that replicates as a large cosmid-like plasmid in *E. coli* and a phage in *M. smegmatis*. To construct the mt3 deletion derivative, a 200 bp DNA fragment containing TM4 Tmp coding sequences lacking mt3 was electroporated into a recombineering strain of *E. coli* and cells recovered in the absence of selection (see *Experimental procedures*). Phasmids isolated from pools of recovered cells were screened by PCR for the presence of a 342 bp deletion and the DNA of one candidate (phAE87Δ*17*mt3) was purified and confirmed by DNA sequencing. The restriction digestion patterns of the mutant are identical to the parent DNA with the exception of the changes expected from the deletion in gene *17*. A similar strategy (Fig. 5) was used to construct a point mutation in TM4 gene *17* that results in the substitution of a glycine residue for the highly conserved tryptophan at the centre of mt3 (amino acid residue 853 in gp17; Fig. 1). Structure of the phAE87*17*W853G mutant was confirmed by sequencing and restriction analysis.

### TM4 Tmp mt3 is not required for phage viability

**Fig. 6:**
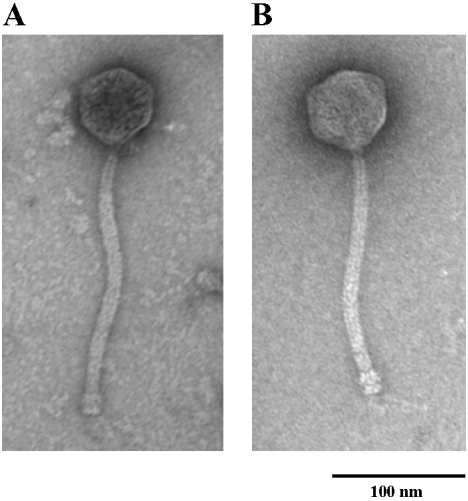
phAE87Δ*17*mt3 is viable but has shorter tails that its parent phage. Electron micrographs of representative particles of phAE87 (A) and phAE87Δ*17*mt3 (B). The average tail length is 200 ± 10 nm for phAE87 and 180 ± 6 for phAE87Δ*17*mt3. The tail lengths of 30 individual phage particles for each phage were measured.

DNA of phAE87, the phAE87Δ*17*mt3 deletion derivative, and the phAE87*17*W853G point mutant were electroporated into *M. smegmatis* and plaques recovered at 30°C (because phAE87 and the mutants are conditionally replicating derivatives of TM4; Bardarov *et al*., 1997). Plaques were recovered from both mutants at similar frequencies to those of their phAE87 parent and no obvious differences in plaque morphology were observed. Thus Tmp mt3 is not essential for growth of TM4. Nevertheless, the relationship between the length of the phage tape measure genes and virion tail lengths is well established, and the 342 bp deletion in TM4 gene *17* is expected to reduce the Tmp by 114 amino acids (Fig. 4A) with a correspondent tail length shortening of about 17 nm (Katsura and Hendrix, 1984). Electron microscopy of phAE87Δ*17*mt3 showed that it possesses tails that are on average approximately 20 nm shorter than those of phAE87, in good agreement with the predicted value (Fig. 6). This not only confirms the role of gp17 in determining TM4 tail length, but also independently validates the construction of the deletion mutant. As expected, the tail length of the phAE87*17*W853G mutant is indistinguishable from that of the parent phage (data not shown).

### TM4 Tmp mt3 is required for efficient infection of stationary phase cells

**Fig. 7:**
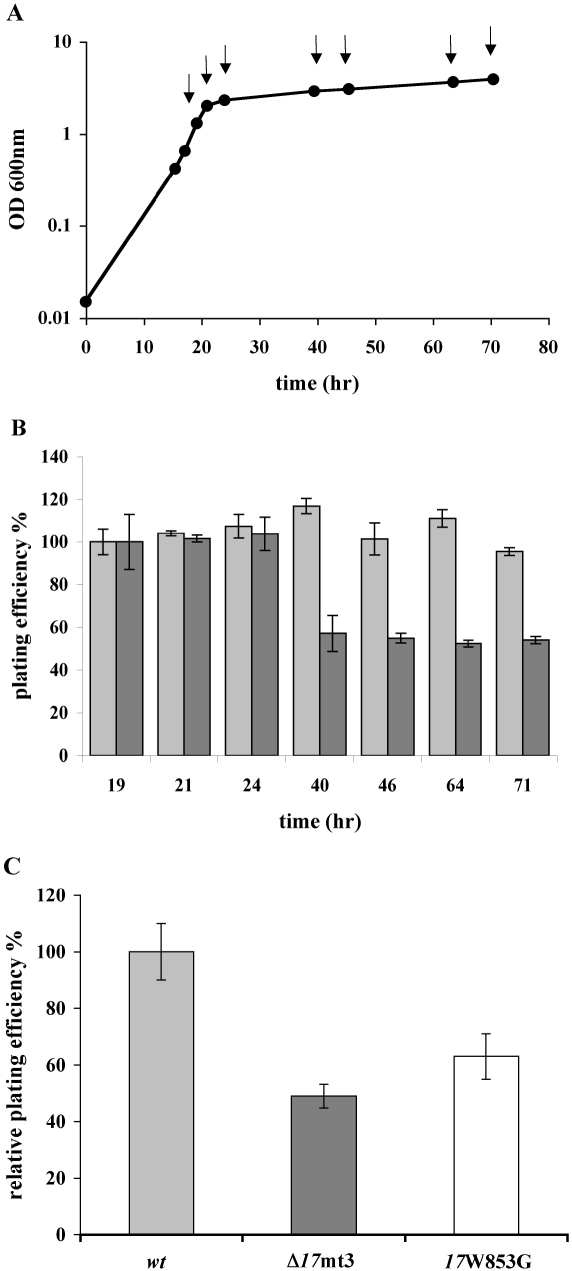
Plating efficiency of wt and mutant phages using cells from different growth phases. A. Growth curve of *M. smegmatis* mc^2^155 at 37°C in 7H9 + ADC. The arrows indicate the time points when cells were removed and used in a plaque assay with phAE87 and phAE87Δ*17*mt3. B. Light grey bars correspond to phAE87 and dark grey bars to phAE87Δ*17*mt3. Plating efficiency was calculated relative to the phage titer at 30°C using cells in late exponential phase of growth (19 h). C. Light grey bar corresponds to phAE87, dark grey bar to phAE87Δ*17*mt3 and white bar to phAE87*17*W853G. Relative plating efficiencies were calculated by comparing the phage titer at 30°C using cells in stationary phase (48 h) relative to the phage titer at 30°C using cells in late exponential phase of growth.

Initial characterization of the Tmp mutants did not reveal any obvious differences from the parent phage strain. Not only are the plaque morphologies identical to phAE87 but the growth patterns in liquid infections are similar and there are no obvious effects of temperature on plating efficiency (Figs S2 and S3). In light of the putative role of MSMEG3721 and MSMEG0642 in cell wall catabolism described above and peptidoglycan hydrolytic activity of TM4 Tmp mt3 – together with previous observations showing that the degree of peptidoglycan cross-linking is significantly increased in stationary phase cells of both *E. coli* and *Bacillus* spp. (Fordham and Gilvarg, 1974; Pisabarro *et al*., 1985; Atrih *et al*., 1999) – we examined the ability of phAE87Δ*17*mt3 to infect *M. smegmatis* at different growth stages of host cells. At all times up to 24 h after inoculation – at which time the cells are just entering stationary phase (Fig. 7A) – the plating efficiencies relative to exponential growing cells of phAE87Δ*17*mt3 and its parent phage are similar (Fig. 7B). However, at all times after 24 h of growth the phAE87Δ*17*mt3 mutant exhibits a reproducible reduction in plating efficiency of about 50% (Fig. 7B). Clearly, the mutant is defective in its ability to infect *M. smegmatis* cells when they are in stationary phase.

To determine that this phenotype results from loss of a functional mt3 in the Tmp and not as a direct consequence of tail shortening (Fig. 6), we analysed the behaviour of phAE87*17*W853G under similar conditions. The phAE87*17*W853G mutant shows a similar phenotype to the deletion mutant although the reduction in infection of stationary cells is more modest than with the deletion mutant (Fig. 7C). Construction of an analogous mutant derivative of the TM4 gp17/MSMEG3721 hybrid protein described above (Fig. 3A) does not completely eliminate activity in a zymogram assay (data not shown), consistent with the intermediate effect on plating efficiency. However, the behaviour of phAE87*17*W853G eliminates the possibility that tail shortening is responsible for the phenotype observed with the deletion derivative.

**Fig. 8:**
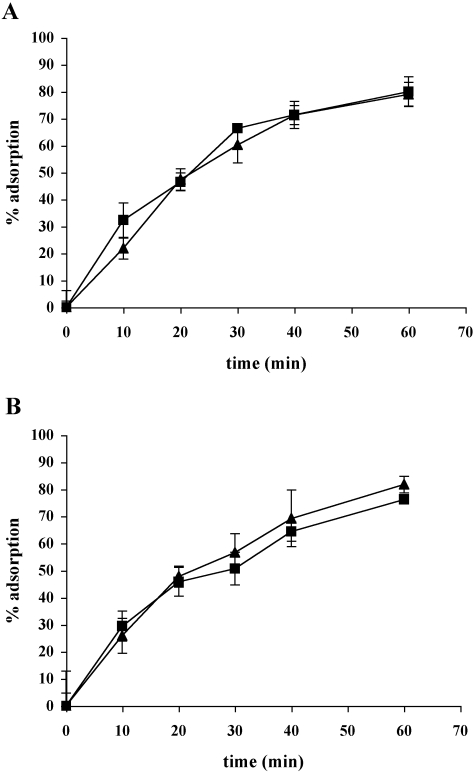
Adsorption assay. Adsorption of phAE8*7* (▪) and phAE87Δ*17*mt3 (▴). A. Adsorption to *M. smegmatis* mc^2^155 cells from exponential phase. B. Adsorption to cells from stationary phase resuspended in spent medium.

The reduction in ability to infect stationary phase cells could result either from a change in the ability to adsorb to host cells, or from a defect in the process of DNA injection; post injection processes (such as virion assembly) seem unlikely because the host cells are growing actively during the formation of bacterial lawns and plaque formation, and the mutants have a normal plaque morphology. However, adsorption assays reveal no differences between phAE87Δ*17*mt3 and phAE87 using either exponentially growing or stationary phase cells (Fig. 8), and thus a loss of adsorption does not account for the defect in infection of stationary phase cells.

**Fig. 9:**
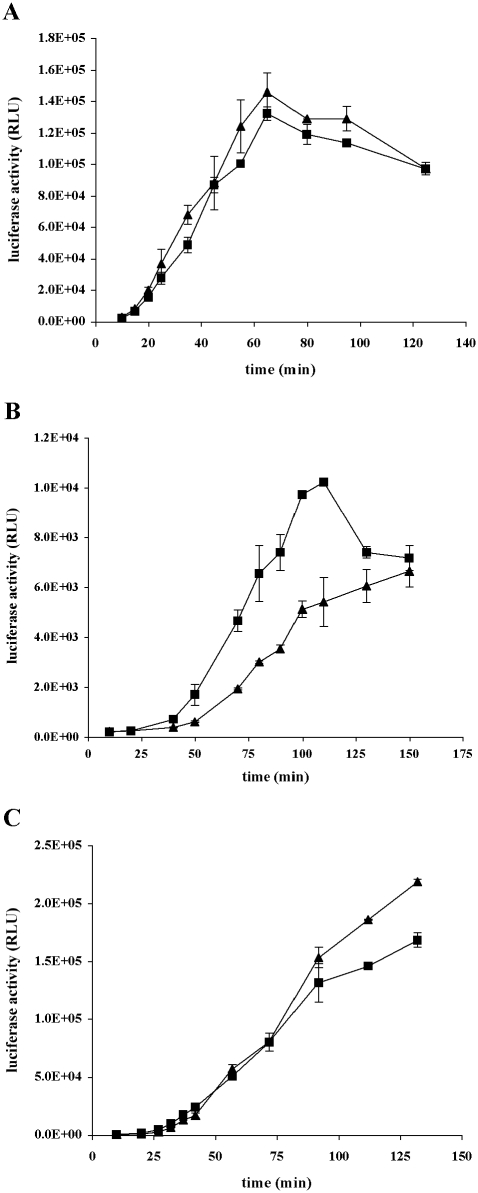
Kinetics of luciferase activity. Kinetics of luciferase activity (relative luciferase units, RLU) after infection with phAE87::*FFlux* (▪) and phAE87Δ*17*mt3::*FFlux* (▴). A. Exponential phase cells. B. Stationary phase cells resuspended in spent medium. C. Stationary phase cells resuspended in fresh medium.

To determine whether DNA injection was altered in the phAE87Δ*17*mt3 mutant we constructed luciferase reporter phage derivatives of phAE87Δ*17*mt3 and phAE87. Upon infection of exponentially growing *M. smegmatis* cells both phages gave similar patterns of light emission related to those reported previously for TM4 reporter phages (Fig. 9A) (Jacobs *et al*., 1993). However, we observed a significant reduction in light output from the phAE87Δ*17*mt3 mutant phage relative to its parent when the host cells were grown to stationary phase and resuspended in spent growth medium such as to maintain a constant cell density. While the overall level of light production from both phages is reduced relative to exponentially growing cells, we observed a reproducible reduction in light output from phAE87Δ*17*mt3 relative to the parent phage (Fig. 9B). For time points up to about 100 min post infection, the phAE87Δ*17*mt3 activities were approximately 50% those of the parent, consistent with the reduction in plating efficiencies shown in Fig. 7. When stationary phase cells were resuspended in fresh media such that they were competent to begin growth – as described previously (Smeulders *et al*., 1999) – we observed a significant amelioration of the mutant defect (Fig. 9C). We note that luciferase reporter phages derived from mycobacteriophage L5 (Sarkis *et al*., 1995) also show a reduction in light output following infection of cells with cell wall alterations (Barsom and Hatfull, 1996).

## Discussion

We have described here a role for the peptidoglycan-degrading activity within the tape measure protein of mycobacteriophage TM4 that facilitates the efficient infection of stationary phase bacterial cells. While we have only demonstrated this activity for the phage TM4, it is plausible that other mycobacteriophages with Tmp's containing mt3 (i.e. Che8 and Omega) may act similarly, in spite of sequence variations within the motif itself (see Fig. 1). As *Micrococcus luteus* Rpf also has peptidoglycan-degrading activity (Telkov *et al*., 2006), it seems plausible that peptidoglycan degradation may be a common activity of all three of the sequence motifs that are located within mycobacteriophage Tmp's.

The presence of mt3 in the TM4 Tmp clearly facilitates infection of cells in stationary phase, and it would not be surprising if this provides a selective advantage to phage propagation in natural environments. However, little is known about the specific growth states of host cells that mycobacteriophages encounter in such environments, although it seems probable that they are more likely to be growing in biofilm communities rather than as exponentially growing cells in a nutritionally rich medium. While the fate of attempts by phage particles to infect non-growing cells is not clear, the data presented here suggest that the peptidoglycan-degrading motif facilitates this process. However, the peptidoglycan-degrading activities of the Rpf-related proteins are also required for stimulating the growth of dormant bacteria, and it is thus plausible that mt3 performs a similar function, in addition to enabling the injection of phage DNA into non-growing cells.

There are many possible reasons to account for the inability of seeing peptidoglycan modifying activity directly from the phage including the amount and renaturation properties of this large protein. However, it is also plausible that proteolytic processing is required to activate the enzymatic activity of this motif, and we note that proteolytic maturation of T4 gp5 is required for full activation of its enzymatic potential (Kanamaru *et al*., 2005). Proteolytic processing is also common among phage Tmp's and may occur at the C-or N-termini, or even lead to loss of an internal segment (Tsui and Hendrix, 1983; Pedersen *et al*., 2000). Processing of TM4 gp17 has not been described in detail although the fate of labelled proteins during phage infection suggests that it is proteolytically processed (Ford *et al*., 1998). Nevertheless, the motif itself does appear to contain peptidoglycan-hydrolysing potential, because it exhibits this activity when incorporated as part of a hybrid protein with MSMEG3721.

The fate of tape measure proteins during an infectious cycle is unclear although they presumably must exit the lumen of the tail in order to provide passage for the DNA into the cell. There are few studies exploring this question, although Roessner and Ihler proposed that the tape measure protein of phage lambda is associated with the membrane and may play a role in forming a transmembrane hole through which the DNA traverses (Roessner and Ihler, 1984). This is an attractive explanation for the role of all tape measure proteins and is consistent with the requirement for peptidoglycan degradation in order to reach the cellular membrane. It has also been proposed (Molineux, 2006) that ejection of the Tmp from the tail lumen could be responsible for transducing a signal from the tail component in contact with the cell surface to the head to initiate DNA ejection. Peptidoglycan hydrolytic activity of mt3 could thus facilitate DNA injection into the host cell.

The observation that TM4 Tmp mt3 is not essential for phage infection is consistent with the finding that the murein hydrolase activities of T4, T7, PRD1 and ϕ6 (Nakagawa *et al*., 1985; Moak and Molineux, 2000; Rydman and Bamford, 2000; Kanamaru *et al*., 2002) are also not required for infection. Interestingly, mutants defective in T7 gp16 exhibit modest defects in either the latent period, lysis time, or the efficiency of plating (Moak and Molineux, 2000), with the greatest effects when cells are entering stationary phase when the peptidoglycan is more highly cross-linked (Pisabarro *et al*., 1985). As phAE87Δ*17*mt3 also shows a defect in the infection of stationary phase cells, this raises the intriguing possibility that the TM4 Tmp is performing a similar role in infection to that of T7 gp16. Remarkably, a substitution of one of the conserved residues in mt3 led to a similar phenotype, corroborating that the phenotype observed was consequence of the lack of the motif in the Tmp and not just a result of a short tail in phAE87Δ*17*mt3. However, construction of the analogous TM4gp17W853G amino acid substitution in the MSMEG3721 hybrid protein did not completely obliterate peptidoglycan hydrolysis in a zymogram (data not shown) and the residual mt3 activity may account for the more modest reduction in plating efficiency (to only 60% in phAE87*17*W853G compared with 50% in phAE87Δ*17*mt3). We note that a different substitution (in the putative catalytic glutamate residue) in the *Micrococcus luteus* Rpf also exhibits activity in a zymogram but is attenuated using other assays (Mukamolova *et al*., 2006). As the zymogram provides only a qualitative measure of muralytic activity, an *in vitro* assay to quantify the exact activity reduction in the mutant proteins and to further characterize the type of activity of mt3 would be helpful. It remains unclear, however, why the defects in phAE87Δ*17*mt3 and phAE87*17*W853G lead to a ∼50% reduction in infection, although it could be due to either a uniform loss in infectivity, or possibly reflect a proposed physiological heterogeneity of stationary phase cells (Blokpoel *et al*., 2005).

While mycobacterial growth state-dependent changes in peptidoglycan cross-linking have yet to be described, we note that this not only occurs in *E. coli* – where murein cross-linking increases at the transition from exponential growth to stationary phase and continues for the next 12 h (Pisabarro *et al*., 1985) and L,D-cross-links are found in the peptidoglycan of stationary phase cells (Templin *et al*., 1999) – but similarly in Gram-positive bacteria such as *B. subtilis* and *Bacillus megaterium* (Fordham and Gilvarg, 1974; Atrih and Foster, 1999). We also note that dilution of stationary phase mycobacterial cells into fresh medium ameliorates the difference exhibited by the phAE87Δ*17*mt3 mutant consistent with the idea that the peptidoglycan is rapidly remodelled in growing cells as shown in *E. coli* (Pisabarro *et al*., 1985). We therefore predict that there are significant changes – either qualitatively and/or quantitatively – in the peptidoglycan of mycobacteria depending on the growth phase of the cells.

The studies reported here provide strong support for the role of a peptidoglycan hydrolase activity of the TM4 Tmp, and we predict that all three of the previously described motifs will play similar roles. However, it is unclear whether those mycobacteriophages that do not appear to harbour one of three motifs are simply at a selective disadvantage to those that do, whether they also have peptidoglycan-hydrolysing activities that have yet to be identified bioinformatically, or whether they possibly naturally infect hosts whose peptidoglycan structures do not prevent barriers to infection, even in stationary phase cells. Peptidoglycan-hydrolysing motifs corresponding to peptidase and transglycosylase activities are located within tape measure proteins of phages that infect other Gram-positive bacteria – including *Gordonia terrae*, *Staphylococcus aureus*, *Lactobacillus* spp., *B. megaterium* and *Mycobacterium vanbaalenii* – suggesting that this is a common strategy employed by phages of Gram-positive bacterial hosts.

## Experimental procedures

### Bacterial strains and growth

*Mycobacterium smegmatis* mc^2^155 was grown at 37°C in Middlebrock 7H9 broth (Difco), containing ADC (2 g l^−1^ Dextrose, 5 g l^−1^ Albumine, 0.85 g l^−1^ NaCl) or 2 g l^−1^ of succinate or acetamide and 0.05% Tween 80. Tween was omitted when cultures were used for phage infection. When appropriate, antibiotics were added at the following concentrations: kanamycin 25 μg ml^−1^, hygromycin 150 μg ml^−1^. *E. coli* strains were grown in L-Broth.

Plasmids used in this study are described in Table 2.

**Table 2:** Plasmids used in this study

Plasmid	Characteristics	Source or reference
pJL37	Shuttle plasmid, Kan^R^, *oriM*, *oriE*, *M. bovis* BCG *hsp60* promoter	Lewis and Hatfull (2000)
pMP1	pJL37 containing MSMEG3721 CDS with the putative SP (NdeI-HindIII)	This work
pMP2	pJL37 containing MSMEG3721 CDS without the putative SP (NdeI-HindIII)	This work
pLAM12	Derivative of pJL37, BCG *hsp60* promoter was replaced with an acetamidase promoter from pSD26	Lab collection
pMP3	pLAM12 containing MSMEG3721 CDS with the putative SP (NdeI-HindIII)	This work
pMH94	Mycobacteria integrative vector, Kan^R^, L5 attP and integrase	Lee *et al*. (1991)
pMP4	pMH94 containing MSMEG3721 CDS with the putative SP under control of acetamidase promoter from pMP3 (XbaI-NheI)	This work
pYUB854	Cosmid, *oriE*, Hyg^R^, *res-hyg-res*	Bardarov *et al*. (2002)
pMP5	pYUB854 containing 5′ and 3′ region of MSMEG3721 and 600 bp of upstream and downstream DNA (XbaI-XmaI and XhoI-BstBI)	This work
pMP6	pYUB854 containing 5′ and 3′ region of MSMEG0642 and 600 bp of upstream and downstream DNA (KpnI-XbaI and XhoI-BglII)	This work
pMP7	pYUB854 containing 5′ and 3′ region of MSMEG6076 and 600 bp of upstream and downstream DNA (AflII-XbaI and HindIII-XhoI)	This work
pPG1	Derivative of pJL37 lacking the BCG *hsp60* promoter	Ghosh *et al*. (2006)
pMP8	pPG1 containing MSMEG3721 gene with the putative promoter and transcription terminator sequences (PCR fragment cloned in BsaAI)	This work
pMP9	pPG1 containing MSMEG0642 gene with the putative promoter and transcription terminator sequences (PCR fragment cloned in BsaAI)	This work
pET-28a	Cloning/inducible expression vector, Kan^R^, N-terminal His Tag	Novagen
pMP10	pET-28a containing MSMEG3721 CDS without the putative SP (NdeI-HindIII)	This work
pMP11	pET-28a containing MSMEG0642 CDS without the putative SP (NdeI-HindIII)	This work
pMP12	pET-28a containing MSMEG3721 TM4 Tmp mt3 (hybrid protein) (NdeI-HindIII)	This work
phAE87	pH101::pYUB328 that fails to replicate at 37°C. PH101 is a *ts* mutant of TM4, Amp^R^	Bardarov *et al*. (1997)
phAE87Δ*17*mt3	pHAE87Δ mt3 in gene *17*	This work
phAE87*17*mt3W4G	phAE87 with a single base change T-G in gene *17* that generates a W4G substitution in Tmp mt3	This work
pYUB556	Cosmid, Kan^R^, *oriE* and *FFlux* under control of BCG *hsp60* promoter	Carriere *et al*. (1997)
pJV24	Kan^R^, *oriM*, Che9c gp59–62 under control of acetamidase promoter	J.C. van Kessel and G.F. Hatfull (submitted)
pGH542	Tet^R^, *oriM*, γδ resolvase under control of BCG *hsp60* promoter	Lab collection

### Cell localization experiments

Plasmids pMP1 and pMP2 were used to transform *M. smegmatis* mc^2^155 electrocompetent cells. Cells were grown in regular media to exponential phase and subjected to differential cell fractioning as described below. *M. smegmatis* mc^2^155 was also transformed with plasmid pMP4 (Table 2) and cells were grown in TH9 media containing succinate (uninduced) or succinate plus acetamide (induced). Cells were grown in 30 ml medium to exponential phase, harvested, and the supernatant recovered. After filtration, 0.015% sodium deoxycholate was added to the supernatant with shaking for 10 min at room temperature; trichloroacetic acid (final concentration 10%) was added and tubes kept for 1 h at 4°C. The pellet was recovered after centrifugation at 4000 *g* for 15 min at 4°C, washed twice with cold acetone and resuspended in phosphate-buffered saline (PBS) (supernatant fraction). The cell pellets were resuspended in 3 ml of PBS containing 1 mM PMSF and sonicated 10 times with 20 s pulses. After centrifugation at 11 000 *g* for 15 min to remove entire cells and insoluble material, the supernatant was centrifuged at 27 000 *g* for 1 h at 4°C. The pellet was resuspended in 300 μl of PBS (cell wall fraction) and the supernatant centrifuged at 100 000 *g* for 1 h at 4°C. The pellet was resuspended in 300 μl of PBS corresponded to the cell membrane fraction and the supernatant was the cytoplasm fraction. Protein concentrations of all fractions were determined using the BIORAD protein estimation kit (cat# 500-0006). Ten micrograms of total protein per sample was loaded on 12% PAGE, blotted onto PVDF membrane and probed with a rabbit anti-MSMEG3721 polyclonal antiserum raised against recombinant MSMEG3721 (1:1000 dilution). Peroxidase-conjugated anti-rabbit IgG (Sigma, A-0545) was used as a secondary antibody (1:10 000 dilution) and detection was carried out using the Western Lightening Reagent (Perkin Elmer) according to the manufacturer's instructions.

### Construction of mutants

The chromosomal copy of MSMEG0642 was interrupted using a specialized transducing phage as described previously, using plasmid pMP6 as the source for allelic exchange (Bardarov *et al*., 2002). To generate MSMEG3721 and MSMEG6076 mutants (MSMEG3721::*res-hyg-res* and MSMEG6076::*res-hyg-res*), we used a recombineering-based system in *M. smegmatis* (J.C. van Kessel and G.F. Hatfull, submitted). Briefly, *M. smegmatis* mc^2^155 was transformed with pJV24, cells were grown in the presence of succinate and recombineering functions were induced for 3 h with acetamide. After that time electrocompetent cells were made and transformed with 100 ng of linearized plasmid pMP5 or pMP7 DNA. After recovery, cells were plated in the presence of kanamycin and hygromycin and incubated for 4 days at 37°C. Ten colonies were checked for gene replacement by colony PCR and one positive clone for each mutant was cured from plasmid pJV24, checked by Southern Blot and used for further studies. To generate the MSMEG3721/MSMEG0642 double mutant (MSMEG0642Δ/3721:: *res-hyg-res*), we transformed MSMEG0642:: *res-hyg-res* with pGH542 that express the γδ resolvase. After recombination between the *res* sites, the Hyg^R^ cassette is removed and lost in subsequent cell divisions, rendering strain MSMEG0642Δ, Hyg^S^. This resultant strain was transformed with pJV24 and after induction of recombineering functions as described above, was transformed with linearized pMP5 to interrupt the chromosomal copy of MSMEG3721. The double mutant strain was cured from plasmid pJV24 checked by Southern Blot and used for further studies. For complementation assays MSMEG0642Δ/3721:: *res-hyg-res* was transformed with plasmids pPG1 (empty vector control), pMP8 or pMP9.

### Minimal inhibitory concentration (MIC) calculations

Wild-type and mutant strains were grown to exponential phase and diluted to an initial OD_600_ of 0.005 in Middlebrock 7H9 broth containing ADC and 0.05% Tween 80 in the presence of different concentrations of vancomycin (0–0.3–0.6–1.25–2.5–5−10 μg ml^−1^). For the plasmid containing strains, kanamycin (25 μg ml^−1^) was also included. Cultures were incubated at 37°C for 48 h; the OD_600_ reached was measured and the percentage of growth for each vancomycin concentration was plotted. The minimal concentration of vancomycin that inhibited growth (MIC) was calculated from the graph (Fig. S1). Experiments were done in triplicate and the standard deviation (SD) was calculated.

### Purification of MSMEG3721 and MSMEG0642 recombinant proteins

N-terminal his-tagged MSMEG3721 and MSMEG0642 without SP were purified after overexpression in *E. coli* BL21 (DE3) (Invitrogen) transformed with plasmids pMP10 and pMP11 respectively. In addition, empty vector pET-28a was also transformed into *E. coli* BL21 (DE3) and prepared in parallel as a negative control. Transformed cells were grown at 37°C to an OD_600_ of 0.6 and protein expression was induced by addition of 0.5 mM IPTG, cells were left overnight at room temperature before harvesting. Cells pellets were resuspended in binding buffer (50 mM Tris-HCl pH 8, 300 mM NaCl, 5% glycerol and 1 mM PMSF) and disrupted by sonication (4 × 15 s). The lysate was centrifuged at 20 000 *g* for 20 min, and the supernatants incubated with 2 ml pre-equilibrated Ni-Agarose for 2 h at 4°C. The matrix was washed with 20 vols of binding buffer, 10 vols of the same buffer containing 10 mM imidazole and 5 vols containing 20 mM imidazole. Elution was done with 4 vols of buffer with 60 mM imidazole. Eluted samples were dialysed twice against binding buffer and a last time in the same buffer but containing 50% glycerol and stored at −20°C.

### Construction of a hybrid protein carrying TM4 Tmp mt3

To construct a MSMEG3721 hybrid protein where the conserved motif was replaced with the Tmp mt3 of TM4, a PCR cloning strategy was used. A PCR product containing gene *17* mt3 and 25 bp of homology to the 5′ and 3′ regions of MSMEG3721 flanking mt3 was generated. Two more individual PCR products were generated corresponding to the 5′ and 3′ region of MSMEG3721 excluding the sequence coding for the conserved motif and the putative SP. The three generated products were used as substrates in a PCR reaction to obtain a full-length amplicon that corresponds to the DNA sequence coding for the hybrid protein. Outside primers (MSMEG3721 Wout/SP **F** and **R**, Table S2) that amplify the whole product and contained restriction sites to clone in pET28 were included in this reaction. After cloning in pET28, pMP12 was generated. Plasmid pMP12 was used to transform *E. coli* BL21 (DE3) (Invitrogen) as described above and a cell extract expressing the hybrid protein was tested for activity in zymogram assay. After expression in pET28 the composition of the hybrid protein will be: 21 amino acids in the N-terminal region (containing the His-tag) (replacing the putative SP – 25 amino acids), 62 amino acids of MSMEG3721, 114 amino acids corresponding to TM4 Tmp mt3 and 22 final amino acids of MSMEG3721.

### Zymogram analysis

Protein samples (5–10 μg) were boiled for 3 min in sample buffer (62.5 mM Tris-HCl pH 6.8, 2% SDS, 20% Glycerol, 5% β-mercaptoethanol, 0.1% Bromophenol Blue) and loaded onto 12% polyacrylamide gels containing 0.1% *Micrococcus luteus* peptidoglycan (Sigma 53243). Gels were cast according to Laemmli (1970) but with only 0.01% SDS. After running, the gel was incubated at 37°C for 16 h in 1% Triton X-100, 25 mM Tris-HCl pH 8.5; washed once in water, stained for 3 h with 0.5% Methylene Blue in 0.01% KOH and destained with water. Peptidoglycan hydrolase activity is detected as a clear zone in a blue background of stained peptidoglycan. Gels not containing peptidoglycan were stained with Coomassie Brilliant Blue. Lysozyme and β-casein used as controls were purchased from Sigma.

### Construction of mutant TM4 phages using recombineering in *E. coli*

*Escherichia coli* strain DY331 carrying a defective λ prophage harbouring the recombination genes *exo*, *bet* and *gam* under the control of temperature-sensitive cI repressor, was used for phAE87 modification. Phasmid phAE87 is a conditional shuttle phasmid vector that contains the TM4 genome and can replicate as a plasmid in *E. coli* and as a phage in mycobacteria at 30°C but not at 37°C. DY331 cells were induced and made electrocompetent as described (Thomason and Oppenheim, 2005). Briefly, 25 ml of cells grown at 30°C to an OD_600_ of 0.6 was induced at 42°C for 15 min. After being chilled on ice for 15 min, cells were washed with ice-cold water three times and resuspended in 500 μl of ice-cold water and used immediately for electroporation. To generate the deletion of mt3 in gene *17* of TM4 genome a 200 bp PCR targeting vector was constructed using a method described previously (Swaminathan *et al*., 2001). Briefly, three primers were designed: a 100-mer oligonucleotide with 50-base homology from either side of the region to be deleted (Recombineering Oligo), and two 72-mer external primers, one forward and one reverse, with 22-base overlaps on either side of the 100-mer (recombineering 50 bp more F and R) (Table S2). Combination-PCR was carried out by mixing 10 ng of the 100-mer and 300 ng of the 72-mers in a 100 μl PCR reaction using the Cloned Pfu DNA polymerase (Stratagene). The resultant PCR product, a 200-mer targeting cassette, was extracted from the gel using the QIAquick Gel Extraction Kit (Qiagen) and reconstituted in sterile water. The PCR product was denaturated at 95°C for 10 min and quick chilled on ice prior to use for electroporation.

Approximately 100 μl of freshly prepared electrocompetent DY331 cells were co-transformed with 500 ng of phAE87 and 200 ng of the 200-mer targeting cassette. Phasmid phAE87 contains a ColE1 origin of replication, and induction of Gam expression in DY331 inactivates RecBCD nuclease. In the absence of RecBCD, ColE1 containing plasmids replicate by a rolling circle mode, and the plasmid converts from monomers to multimers. By co-electroporating the *recA*^–^ strain DY331 with phAE87 and the linear targeting cassette recombinant plasmid monomers can be readily selected and isolated (Yu *et al*., 2000). After electroporation cells were resuspended in 1 ml of Luria–Bertani medium and recovered at 30°C for 2 h. Cells were diluted and plated in 1-ml-deep 96 well plates at about 5–10 cells per well. The cells were grown at 30°C for about 24 h in the presence of carbenicillin 50 μg ml^−1^ and tetracycline 12.5 μg ml^−1^. The number of cells per well was determined by plating serial dilutions of electroporated cells on agar plates. After 24 h, phasmids from each pool of cells were isolated using a Qiagen BioRobot™ 9600 nucleic acid purification system and analysed by PCR. One of the samples containing phAE87Δ*17*mt3 was used to transform XL-Blue competent cells. Twenty-five colonies were analysed to find a clone containing only the modified phasmid. Phasmid mutant phAE87Δ*17*mt3 was checked by restriction analysis and sequencing. In all the analysed phasmids (about 10) the deletion occurred correctly, eliminating precisely the region corresponding to mt3.

A similar strategy was used to generate a single base change in phAE87, T12741G that changes a TGG for a GGG codon (W853G) in gene *17*. In this case, two complementary single-strand 100-mer oligonucleotides (Recombineering Oligo T→G + and –) in which a T in the original sequence was replaced by a G were used as substrates. Recombination was also performed in *E. coli* without selection and phasmids isolated from pools of recovered cells screened by an allele-specific PCR amplification called the mismatch amplification mutation assay (MAMA-PCR) (Cha *et al*., 1992). A detection primer containing a two-base mismatch to the wild-type sequence but only a penultimate base mismatch to the point mutation at its 3′ end (MAMA-PCR **R**) was used along with a forward primer (MAMA-PCR **F**). The PCR condition included denaturation for 4 min at 94°C followed by 40 cycles of 94°C for 15 s and 77°C for 1 min (a common annealing and extension temperature) and a final extension at 72°C for 7 min. These primers amplify a 568 bp product. Two positive clones were used to transform XL-Blue cells and in 20 clones of each the presence of a single phasmid containing the desired mutation was checked by a second round of MAMA-PCR and corroborated by restriction analysis of a 939 bp PCR product amplified from the mutated region. The base change created a new Pvu I site absent in the original sequence, so after restriction digestion the mutated PCR product generates two fragments of 528 and 409 bp. Phasmid mutant phAE87*17*W853G was checked by restriction analysis and sequencing. Three clones, one of phAE87, one of phAE87Δ*17*mt3 and other of phAE87*17*W853G, were used to transform *M. smegmatis* in order to recover plaques and generate phage stocks for use in further experiments.

### Construction of phAE87::FFlux phages

To construct phAE87 and phAE87Δ*17*mt3 carrying a luciferase gene, plasmid pYUB328 was replaced for pYUB556 containing the luciferase cassette. Plasmid pYUB556 is a derivative of pUB216, where Amp^R^ was replaced for a Kan^R^ cassette (Jacobs *et al*., 1993). Briefly, phAE87 and phAE87Δ*17*mt3 were digested with NotI and ligated to pYUB556 cut with the same enzyme. About 100 ng of the ligation mix was packaged using an *in vitro* λ-packaging reaction (Gigapack III XL-7, Stratagene). After transducing *E. coli* HB101 and plating the transductants on selective media containing kanamycin, phasmid DNA was prepared from 10 colonies of each transduction and checked by restriction analysis. Two clones, one of phAE87 and other of phAE87Δ*17*mt3, were used to transform *M. smegmatis* in order to recover plaques and generate a phage stock of phAE87::*FFlux* and phAE87Δ*17*mt3::*FFlux* to use in further experiments.

### Luciferase assays

Luciferase assays were performed as already described (Sarkis *et al*., 1995). Cells were grown to exponential phase or stationary phase and diluted to an initial OD_600_ of 0.1. When stationary phase cells were used, 5 ml of culture was centrifuged and resuspended in the same volume of either fresh medium (7H9 broth containing ADC, without Tween) or filtered spent medium and subsequently diluted to an initial OD_600_ of 0.1 in fresh or spent medium respectively. Assays were initiated by addition of 0.1 ml of phages (10^10^ pfu ml^−1^) to 0.9 ml of cells (10^7^ cfu ml^−1^, moi: 100). Cultures were incubated standing at 37°C for 15 min and then with agitation. At various time points, a 50 μl aliquot was removed after vortexing the sample for about 10 s. The aliquot was placed in a polystyrene cuvette (Monolight 2050–10) and light output was measured for 20 s after the injection of the substrate [100 μl of 1 mM D-luciferin (BD Biosciences, 556876) in 0.1 M sodium citrate, pH 5] in an Analytical Luminescence Laboratory Monolight 2010 single injector luminometer.

### Adsorption assays

About 1.5 × 10^8^ cells from exponential and stationary phase were incubated with 7 × 10^3^ phages (moi: 0.00005) at 30°C either in fresh or spent media in 3 ml final volume. At the indicated time points, 120 μl of aliquots was removed, after vortexing for 5 s, and centrifuged for 1 min at 13 000 rpm to remove cells. One hundred microlitres of the supernatant was mixed with a 100 μl of exponential cells, incubated at 30°C for 30 min and mixed with 3 ml of TH10 Top Agar that was quickly poured onto a pre-warmed TH10 plate. Plaques were visible after incubation at 30°C for 48 h.

### Bioinformatic analyses

Identification and comparison of conserved sequence motifs were performed using ProDom (http://prodom.prabi.fr/prodom/current/html/home.php), and signal sequences were identified using Signal P 3.0 (http://www.cbs.dtu.dk/services/SignalP) (Nielsen and Krogh, 1998; Bendtsen *et al*., 2004).

## Supplementary material

The following supplementary material is available for this article online:

Fig. S1. Growth response of *wt* and mutant strains to vancomycin.

Fig. S2. Plating efficiency of phAE87 and phAE87Δ17mt3 at different temperatures.

Fig. S3. phAE87 and phAE87Δ17mt3 infection in liquid cultures at different temperatures.

Table S1. Oligonucleotides used in this study.

This material is available as part of the online article from http://www.blackwell-synergy.com
