# Supplementary material for: A peptidoglycan hydrolase motif within the mycobacteriophage TM4 tape measure protein promotes efficient infection of stationary phase cells
Source: Mol Microbiol. 2006 Dec 2;62(6):1569–85. doi: 10.1111/j.1365-2958.2006.05473.x (PMC1796659; doi:10.1111/j.1365-2958.2006.05473.x)
Supplement: Fig. S1. — Growth response of wt and mutant strains to vancomycin. [file mmi0062-1569-Figs-Tables.pdf]

A peptidoglycan hydrolase motif within the mycobacteriophage TM4 tape measure protein promotes efficient infection of stationary phase cells.

Mariana Piuri and Graham F. Hatfull

Pittsburgh Bacteriophage Institute and
Department of Biological Sciences
University of Pittsburgh
Pittsburgh, PA 15260
Tel: (412) 624 4350
FAX: (412) 624 4870
Email: gfh@pitt.edu

Fig. S1. Growth response of *wt* and mutant strains to vancomycin.
The mean of three independent experiments and the standard deviation are shown.
M. smegmatis mc2155 wt (◆); MSMEG0642Δ/3721:: *res-hyg-res* (▲); MSMEG3721:: *res-hyg-res* (●); MSMEG0642:: *res-hyg-res* (■); MSMEG6076:: *res-hyg-res* ( )

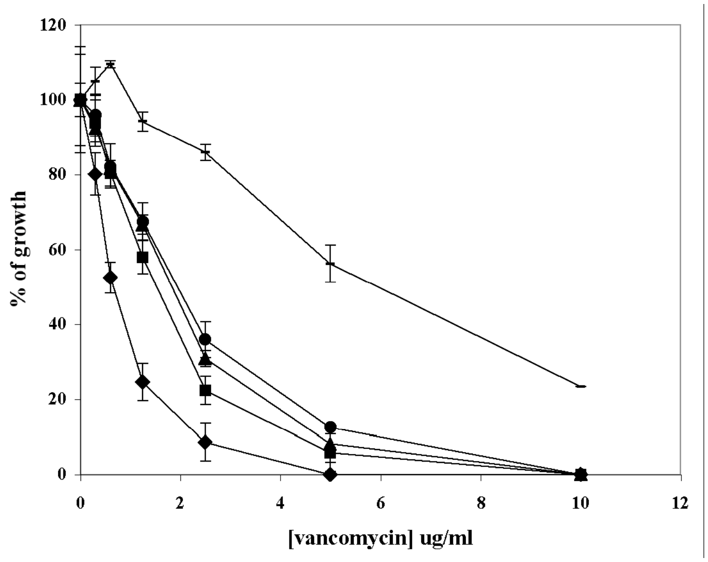

Fig. S2. Plating efficiency of phAE87 and phAE87Δ17mt3 at different temperatures.
Light gray bars correspond to phAE87 and dark gray bars to phAE87Δ17mt3. Plating efficiency was calculated relative to the phage titer at 30°C using cells in late exponential phase of growth (19 hr).

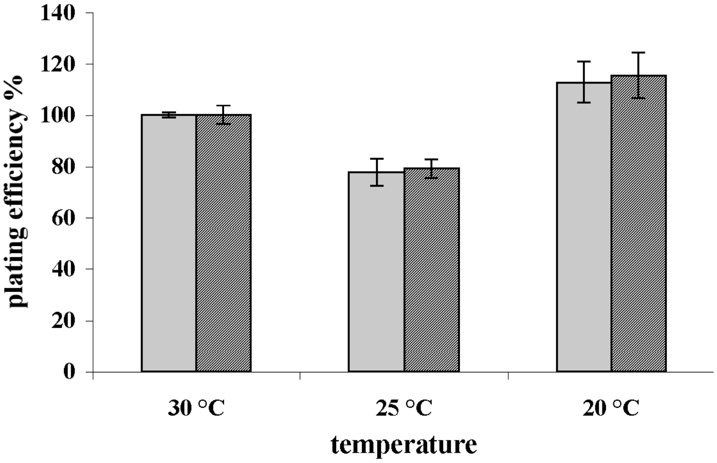

Fig. S3. phAE87 and phAE87Δ17mt3 infection in liquid cultures at different temperatures.
M. smegmatis mc2155 was grown at: A, 30°C; B, 25°C; C, 20°C to an OD600nm of about 0.3 and infected with phAE87 (squares) and phAE87Δ17mt3 (triangles) at a m.o.i = 5. Infected cultures were incubated with agitation at the same temperature and the absorbance was monitored during time in order to monitor cell lysis.

A

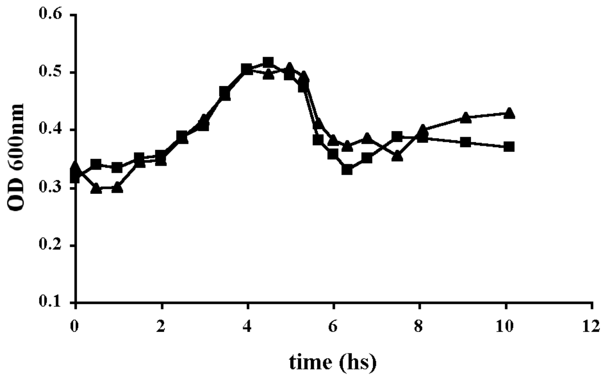

B

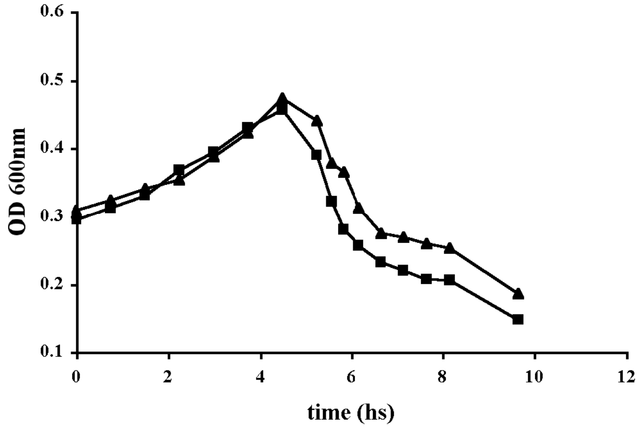

C

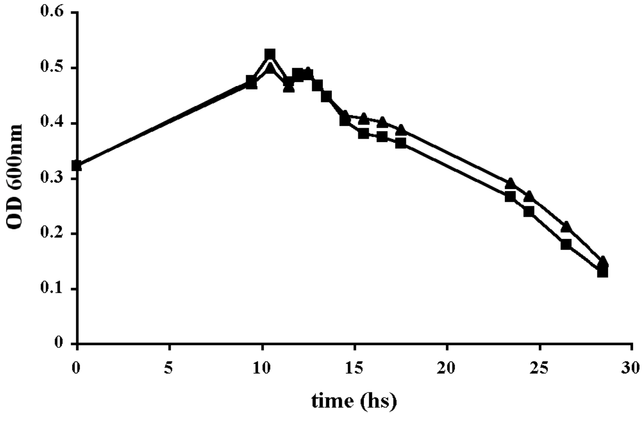

Table S1: Oligonucleotides used in this study

Primer name	Sequence	Comment
MSMEG3721 Wout/SP **F**	GGAATTCCATATGGCGATGTTCTATGCGCAGGGC	Includes NdeI site to clone in pet28a and pJL37
MSMEG3721 W/SP **F**	GGAATTCCATATGGTGGGTGGTATCCGGTTGGCA	Includes NdeI site to clone in pJL37 and pLAM12
MSMEG3721 **R**	TGATCCAAGCTTTCAGTTCGCCAAAGTCTTTGAATTCAG	Includes HindIII site to clone in pet28a, pJL37 and pLAM12
MSMEG0642 Wout/SP **F**	GGAATTCCATATGGAGCCGGACATCAAACCCGC	Includes NdeI site to clone in pet28a
MSMEG0642 **R**	TGATCCAAGCTTTCAGCGCAGGTAGACCTCGCC	Includes HindIII site to clone in pet28a
MSMEG3721 5' **F**<br>MSMEG3721 5' **R**	AAATTTCCAAGCTTACATGCACCATCGTCGATAC<br>GGAATTCCCATATGGATCGTGTTGACCTGCAGAC	To amplify 5' region of MSMEG3721 and 600 bp of upstream and downstream DNA flanking region to clone in pMOS Blue and subclone in pYUB854 (XbaI-XmaI)
MSMEG3721 3' **F**<br>MSMEG3721 3' **R**	TTAGGGAACTCGAGGCCAACGCGAAACGCTGGGGC<br>AAATTCGAACGTTTTCTGCTGGGCGGCCGG	To amplify 3' region of MSMEG3721 and 600 bp of upstream and downstream DNA flanking region to clone in pYUB854 (XhoI-BstBI)
MSMEG0642 5' **F**<br>MSMEG0642 5' **R**	CGGGGTACCCCGGCTTGCGGGAGACGTTC<br>CTAGTCTAGAGAGTTCCATACCCGGCACGG	To amplify 5' region of MSMEG0642 and 600 bp of upstream and downstream DNA flanking region to clone in pYUB854 (KpnI-XbaI)
MSMEG0642 3' **F**<br>MSMEG0642 3' **R**	CCGCTCGAGCCGAACCCGAGCAGCCCAGC<br>GGAAGATCTCCAGCAGCATCCCCACCTGG	To amplify 3' region of MSMEG0642 and 600 bp of upstream and downstream DNA flanking region to clone in pYUB854 (XhoI-BglII)
MSMEG6076 5' **F**<br>MSMEG6076 5' **R**	AATTTCTTAAGAACCGGGGAACACCGACTCGG<br>GCTCTAGAACCGGTACGACGCCGGGAT	To amplify 5' region of MSMEG6076 and 600 bp of upstream and downstream DNA flanking region to clone in pYUB854 (AflII-XbaI)
MSMEG6076 3' **F**<br>MSMEG6076 3' **R**	TGATCCAAGCTTAACGCCGCTGCCGGACACA<br>AACCGCTCGAGCAGGCGGGACGGTCGACG	To amplify 3' region of MSMEG6076 and 600 bp of upstream and downstream DNA flanking region to clone in pYUB854 (HindIII-XhoI)
MSMEG3721 **F** comp<br>MSMEG3721 **R** comp	ACCTGCATCCGGACGGGCTG<br>GTCCCCCCTTGGACGCACCG	To amplify MSMEG3721 including the putative promoter and transcription terminator region, to clone in pPG1
MSMEG0642 **F** comp<br>MSMEG0642 **R** comp	ACAGCGCCACGATGCGGACTGT<br>ACAGCGCCACGATGCGGACTGT	To amplify MSMEG0642 including the putative promoter and transcription terminator region, to clone in pPG1
3721 for 5'region **R**	GGACTCGGGTGCGGCGCC	Used with MSMEG3721 Wout/SP **F** to amplify the 5' region of MSMEG3721 CDS with out the conserved motif
3721 for 3' region **F**	ACAAATGGCGGCGGTTACCCTAC	Used with MSMEG3721 R to amplify the 3' region of MSMEG3721 with out the conserved motif
gp17 mt3 **F** (3721 5' region)	**CCGGGCGCCGCACCCGAGTCC**GGCCTGGCGTCCGAGCAG	To amplify *gene17 mt3*, bold letters correspond to the flanking homology to the 5' and 3'region of MSMEG3721
gp17 mt3 **R** (3721 3' region)	**AGGGTAACCGCCGCCATTTGT**CACGTGCACGTGATCCATGTGG	
Recombineering Oligo	TGGGAGCTGATTAGGGCGCTGCCGAGGTTCGCTGG<br>CGGCGGCCTGGCCGCGACGTCGAAGGGCGGCAAG<br>CCGAAGGGCGGCGTGGACACCGCACCGGCGGGC	Contains 50 bp of homology to each side of the deletion of motif 3 in gene *17* of TM4
Recombineering 50bp more **F**<br>Recombineering 50bp more **R**	GCCCGCAATCTGCCGCTGCTGCAGGCGATCAACTCCG<br>GTGCGCCGCTGTGGGAGCTGATTAGGGCGCTGCCG<br>CACCCCCACGCCGTCGAGGCCGCTCACGTTGACGCCA<br>GACGGCAAAGACAGGCCCGCCGGTGCGGTGTCCAC	To increase the length of homology in the recombineering target vector
gene *17* recomb **F**<br>gene *17* recomb **R**	AAGATGACGTTCACCGTTCC<br>GTGTGCTTCTTACCCTTGAG	To map motif 3 deletion in gene *17*
Recombineering Oligo T G +<br><br>Recombineering Oligo T G -	TGCAGTTCCTTATGAAGAACGCCGACGCGCTCGGCGTCGAGT<br>ACACGATC**G**GGCGGCAGACCTACCGCAACACCTCGGGGCAGT<br>CGAACCTCATGGAGGAC<br>GTCCTCCATGAGGTTCGACTGCCCCGAGGTGTTGCGGTAGGTC<br>TGCCGCC**C**GATCGTGTACTCGACGCCGAGCGCGTCGGCGTTC<br>TTCATAAGGAACTGCA	Contains 50 bp of homology to each side of the single base change T4G in gene *17* of TM4
Scree MAMA-PCR **R**<br>Scree MAMA-PCR **F**	CCACAACGCCATCCAGACGGCG**CG**<br>CGGGCTGGCCGACAAGATGACG	For screening of clones containing a T G base change using MAMA-PCR.
